# Theoretical Modeling of a Bionic Arm with Elastomer Fiber as Artificial Muscle Controlled by Periodic Illumination

**DOI:** 10.3390/polym17152122

**Published:** 2025-07-31

**Authors:** Changshen Du, Shuhong Dai, Qinglin Sun

**Affiliations:** School of Engineering and Mechanics, Liaoning Technical University, Fuxin 123000, China; changshendu@yeah.net (C.D.); sunqinglin_0420@163.com (Q.S.)

**Keywords:** dynamic model, forced oscillation, liquid crystal elastomer, light-fueled, bionic arm, periodic illumination

## Abstract

Liquid crystal elastomers (LCEs) have shown great potential in the field of soft robotics due to their unique actuation capabilities. Despite the growing number of experimental studies in the soft robotics field, theoretical research remains limited. In this paper, a dynamic model of a bionic arm using an LCE fiber as artificial muscle is established, which exhibits periodic oscillation controlled by periodic illumination. Based on the assumption of linear damping and angular momentum theorem, the dynamics equation of the model oscillation is derived. Then, based on the assumption of linear elasticity model, the periodic spring force of the fiber is given. Subsequently, the evolution equations for the *cis* number fraction within the fiber are developed, and consequently, the analytical solution for the light-excited strain is derived. Following that, the dynamics equation is numerically solved, and the mechanism of the controllable oscillation is elucidated. Numerical calculations show that the stable oscillation period of the bionic arm depends on the illumination period. When the illumination period aligns with the natural period of the bionic arm, the resonance is formed and the amplitude is the largest. Additionally, the effects of various parameters on forced oscillation are analyzed. The results of numerical studies on the bionic arm can provide theoretical support for the design of micro-machines, bionic devices, soft robots, biomedical devices, and energy harvesters.

## 1. Introduction

In recent years, a great number of bionic structures that utilize active materials including liquid crystal elastomers (LCEs) [1,2,3], piezoelectric materials [4,5], nylon [6], hydrogels [7,8,9], and ionogels [10] as power units have attracted wide attention [11,12,13,14]. These bionic machines are energized by light [15,16,17], heat [18,19], electricity [20,21], magnetism [22,23], etc., driven by environmentally induced deformation stresses, and can perform a range of functions such as swimming [24], floating [25], fluttering [26], striking [27], climbing [28], and crawling [23]. Additionally, there are bionic machines that are able to realize complex motions such as jumping [29,30], curling [31], tumbling [32], and flying [33] under the control of periodic illumination, which greatly expands the applications of bionic machines. These machines utilize environmental actuation and have the advantages of a simple structure, no need for electronics, and high intelligence [34,35,36].

Among a variety of stimuli, near-infrared light is a kind of stimulus with unique advantages [36,37,38], such as no chemical pollution, easily available in multiple ways, and widespread existence. Based on photothermal-responsive LCE materials, systems with various movement mechanisms were constructed, including a large deformation mechanism [39], an orthogonal response mechanism [19], a self-shading mechanism [40], an adjustable energy barrier mechanism [41], and a photothermal surface tension gradient mechanism [42]. Based on these motion mechanisms, a variety of motion modes are realized, such as eversion/inversion [6], winding [16], bending [17,34], rolling [43,44], oscillating [45], rolling back [41], jumping [46], and coupled vibration [47,48]. However, most of these works focus on experimental studies, and there is a dearth of theoretical studies on periodic oscillations under the control of periodic illumination, which limits the development and application of bionic machines.

LCE has been extensively proven to be a highly suitable material for use as the actuators of artificial muscles [49,50,51], which has exhibited a remarkable contractile strain of 50% to 80% in response to light or thermal stimuli [52] and has the ability to recover quickly [49]. Due to these advantageous properties, LCE has been extensively explored in the fields of soft robotics and active deformation structure design and fabrication [53,54,55]. For instance, He et al. [56] employed the electrospinning technique to create an LCE microfiber with potential applications as an artificial muscle; Roach et al. [57] utilized three-dimensional (3D) printing to produce an LCE fiber that can be used in smart textiles; Zhou et al. [19] devised an jumpable LCE soft robot by introducing an orthogonal response mechanism. These studies have amply demonstrated that bionic machines utilizing the LCE as an artificial muscle can better mimic animal locomotion in form and function.

The work of He et al. [56] demonstrated that driving bionic machines through periodic illumination to achieve controllable complex motions is an excellent strategy to expand the application scenarios of bionic machines. In this paper, we innovatively establish the theoretical model of a bionic arm with an LCE fiber as artificial muscle. The bionic arm can achieve controllable periodic oscillation under periodic illumination, much like the motion of a human arm. To elucidate the complex dynamics of the periodic oscillation, explain its motion mechanism, explore its control methodology, and offer theoretical support for its applications, we first derive the dynamic equations of the bionic arm oscillation based on the assumption of linear damping and angular momentum theorem. Then, based on the assumption of a linear elasticity model, the periodic spring force of the fiber is given. Subsequently, using the dynamic LCE model developed by Nägele et al. [58], the evolution equations for the *cis* number fraction within the LCE fiber are developed, and consequently, the analytical solution for the light-excited strain is derived. Following that, the dynamics equation is numerically solved by using the classical fourth-order Runge–Kutta method, and the evolution of the *cis* number fraction within the LCE fiber during an illumination cycle and the mechanism of the controllable periodic oscillation is elucidated. Subsequently, this research delves into the oscillation behavior of the bionic arm under different illumination conditions. Finally, the effects of various parameters on the oscillation are quantitatively analyzed. The bionic arm using LCE fiber as an artificial muscle has the advantages of a simple structure, electronics-free, ambient-powered, and rapid response, all of which make it highly practical in the design of micro-machines, bionic devices, soft robots, biomedical devices, and energy harvesters.

## 2. Theoretical Model and Formulation

This section sketches a dynamic model of the forced vibration of a bionic arm controlled by periodic illumination, which consists of a brachium, a forearm, a joint, a mass block, and an LCE fiber as artificial muscle. Based on the dynamic LCE model and the angular momentum theorem, the governing equations of the forced vibration of the bionic arm are derived and the analytical solutions of the equations are solved. Following that, the evolution of the *cis* number fraction within the LCE fiber is elucidated. Finally, the equations are nondimensionalized by defining dimensionless parameters.

### 2.1. Formulation of Light-Powered Forced Vibration of the Bionic Arm

**Figure 1:**
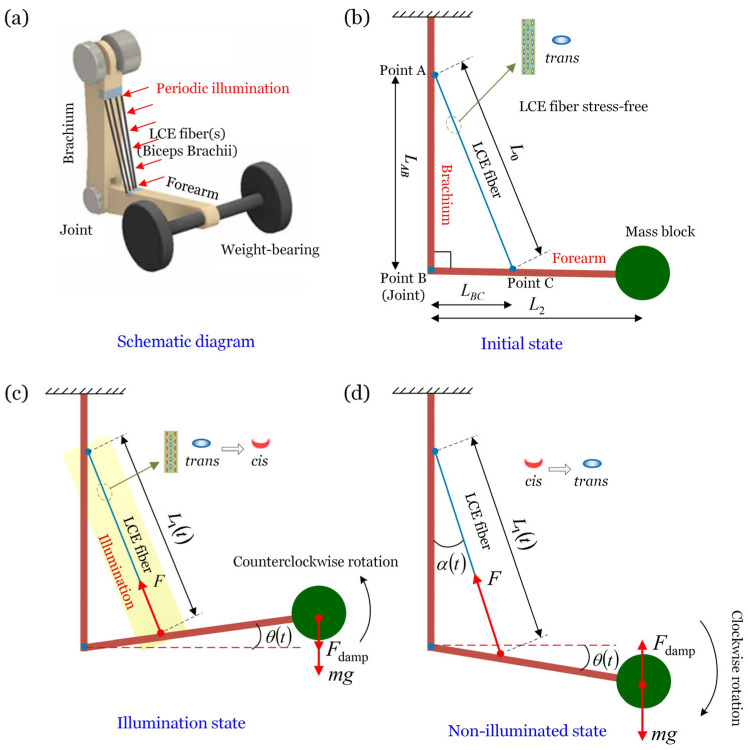
Schematic diagram of the bionic arm using an LCE fiber as artificial muscle controlled by periodic illumination. (**a**) Schematic diagram; (**b**) Initial state; (**c**) Illuminated state; (**d**) Non-illuminated state. The rotation angle is positive in a counterclockwise direction from the horizontal line. Periodic illumination leads to the periodic contraction and relaxation of the fiber, driving the bionic arm to oscillation periodically.

In this section, the dynamic model and governing equations for the bionic arm’s forced oscillation are derived. Figure 1a sketches the dynamic model, which consists of a brachium, a forearm, a joint, a mass block, and an LCE fiber as an artificial muscle, with the model being controlled by periodic illumination. First, the brachium is fixed vertically, and the brachium and forearm are articulated through a hinged joint denoted as point B. Following that, the forearm is adjusted to the horizontal position, while the fiber is kept straight and stress-free. Finally, both ends of the LCE fiber are fixed on the brachium and forearm, respectively, as shown in Figure 1b. The original length of the fiber in the stress-free state is denoted by $L_{0}$, and the connection points are recorded as point A and point C, respectively. The lengths AB and BC are represented by $L_{AB}$ and $L_{BC}$, respectively. It is worth noting that the ΔABC is a right triangle at the initial state. Finally, a mass block with a mass of m is fixed at the end of the forearm, and the straight-line distance between the center of mass block and point B (joint) is $L_{2}$.

When the LCE fiber is not illuminated, the liquid crystal molecules are in trans configuration [58], with the molecular chains in a straightened state, and the fiber is in a stress-free state, as shown in Figure 1b. Then, taking the state of the system in Figure 1b as the initial state, releasing the mass block and exposing the fiber to near-infrared light. Under this illumination, a portion of liquid crystal molecules absorb photons and undergo isomerization from *trans* to *cis* [58]. This microscopic configuration change leads to an increase in the arrangement density between molecular chains, which macroscopically manifests as the fiber’s length contracting from $L_{0}$ to $L_{1}$, as shown in Figure 1c. Furthermore, the shrinking of the fiber leads to an increase in the spring force $F\left(t\right)$ of the fiber. The forearm and mass block are driven by the spring force to rotate clockwise around point B, and the angle of rotation is recorded as $\theta \left(t\right)$, as shown in Figure 1c. The rotation angle is measured from the horizontal line and is positive in the clockwise direction. The shaded area in Figure 1c represents the illumination area. When the LCE fiber is in the non-illuminated state, a portion of the liquid crystal molecules changes from *cis* to *trans* [59], which results in partial recovery of the light-induced contraction and a corresponding reduction in spring force. As a result, the forearm and mass rotate counterclockwise around the point B under the effect of gravity, as shown in Figure 1d. Periodic illumination leads to periodic shrinkage and recovery of the fiber, which drives the periodic oscillation of the bionic arm. We will describe this dynamic process in more detail in Section 3.2.

We estimated the standard values for the dimensions and mass of each component of the bionic arm based on previous studies and empirical data [45,56], as listed in Table 1. By neglecting the bending deformation of the brachium and the forearm, the motion of the bionic arm can be simplified to a one-dimensional rotation. Compared to the mass of the mass block m, we further neglected the masses of both the fiber $m_{\mathrm{fi}}$ and the forearm $M_{\mathrm{fo}}$, thereby reducing model parameters and further simplifying the model. We further assume that the joint is perfectly smooth, and the system damping originates from air resistance. Furthermore, since the oscillation of the bionic arm is small-amplitude and low-frequency, the damping torque is assumed to be linearly proportional to the angular velocity $d\theta \left(t\right)/dt$. The angular momentum theorem for the system about joint B during forced oscillation dictates that the following equation holds at any time

(1) $$mL_{2}^{2}\frac{d^{2}\theta }{dt^{2}}=mg\cos\theta \left(t\right)L_{2}-F\left(t\right)\cos\alpha \left(t\right)L_{BC}-c\;.\;\frac{d\theta }{dt}L_{2}\;.\;L_{2},$$

where g refers to gravitational acceleration, $\alpha \left(t\right)$ is the angle of the fiber in the vertical direction, and c denotes the damping coefficient, $F\left(t\right)$ is the force exerted by the fiber on the forearm, which is considered equal to the spring force of the fiber. In Equation (1), the polynomial on the right-hand side represents the total torque around joint B, including the spring torque, gravitational torque, and damping torque. The term on the left-hand side represents the rate of change of angular momentum with respect to time.

The length of the LCE fiber at any moment during the oscillation is denoted by $L_{1}\left(t\right)$. It is assumed that the brachium, the forearm, and the fiber are in the same plane, and according to the cosine theorem, $\cos\alpha \left(t\right)$ can be expressed in terms of the length of the fiber $L_{1}\left(t\right)$ as

(2) $$\cos\alpha \left(t\right)=\frac{L_{BC}^{2}+L_{1}^{2}\left(t\right)-L_{AB}^{2}}{2L_{BC}L_{1}\left(t\right)}.$$

Similarly, the length $L_{1}\left(t\right)$ can be expressed in terms of the rotation angle $\theta \left(t\right)$ as

(3) $$L_{1}\left(t\right)=\sqrt{L_{AB}^{2}+L_{BC}^{2}+2L_{AB}L_{BC}\sin\theta \left(t\right)}.$$

As shown in Figure 1b, there exists in the initial state $L_{0}=\sqrt{L_{AB}^{2}+L_{BC}^{2}}$. In studying the vibration of the bionic arm, we take into account the combined effects of elastic deformation and light-excited contraction deformation. Naciri et al. [50] experimentally demonstrated that the stress–strain relationship for LCE fiber remains nearly linear within a 20% strain range. Therefore, we make the assumption that the spring force $F\left(t\right)$ of the fiber is directly proportional to the elastic deformation, i.e.,

(4) $$F\left(t\right)=k\left[L_{1}\left(t\right)-L_{0}-\varepsilon_{L}\left(t\right)L_{0}\right],$$

where $L_{0}=\sqrt{L_{AB}^{2}+L_{BC}^{2}}$, k denotes elastic constant of the fiber, and $\varepsilon_{L}\left(t\right)$ refers to light-excited contraction strain. It can be concluded from Equation (4) that the spring force $F\left(t\right)$ of the fiber increases with the increase in the shrinkage strain $\varepsilon_{L}\left(t\right)$ (the absolute value decreases). Moreover, it is worth noting that, when the deformation of the fiber is greater than 20%, nonlinear constitutive relations such as the Ogden model [49] need to be introduced.

### 2.2. Dynamic LCE Model

This section focuses on the dynamic of the light-excited contraction strain and the *cis* number fraction within the fiber. The azobenzene liquid crystal molecules can be transformed from straight *trans* to curved *cis* under the irradiation of near-infrared light, which is macroscopically manifested as a shrinkage deformation of LCE materials. Consider that the contraction strain $\varepsilon_{L}\left(t\right)$ of the fiber induced by light is influenced by the number of bent *cis*-azobenzene liquid crystal molecules in the fiber. For simplicity, we assume that there is a linear relationship between light-excited contraction strain and the *cis* number fraction within LCE fiber, i.e.,

(5) $$\varepsilon_{L}\left(t\right)=C_{0}\phi \left(t\right),$$

where $C_{0}$ represents the light-excited contraction coefficient of the LCE fiber, and $C_{0}<0$. Combined with Equation (4), it can be concluded that when the fraction coefficient $C_{0}$ increases (absolute value decreases), the spring force $F\left(t\right)$ of the fiber decrease. To compute the contraction strain $\varepsilon_{L}\left(t\right)$ of the fiber induced by light, we first need to calculate the *cis* number fraction $\phi \left(t\right)$.

In a previous study, Yanlei et al. [60] demonstrated through experiments that the transition from *trans* to *cis* in liquid crystal molecules can be triggered by near-infrared light with wavelengths below 400 nm. Nägele et al. [58] proposed that the proportion of *cis*-isomers was influenced by thermal excitation from trans to *cis* states, light-induced trans to *cis* isomerization, and the thermal relaxation from *cis* to *trans* states. Additionally, it was noted that light-induced isomerization plays a more significant role compared to thermal excitation, so that thermal excitation effect is neglected in the following study. The governing equation for the evolution of the *cis* number fraction $\phi \left(t\right)$ can be given as

(6) $$\frac{\partial \phi \left(t\right)}{\partial t}=\eta_{0}I_{0}\left[1-\phi \left(t\right)\right]-\frac{\phi \left(t\right)}{T_{0}},$$

where $I_{0}$ represents the light intensity, $T_{0}$ denotes the time of thermal relaxation, and $\eta_{0}$ refers to the light-absorption constant. The *cis* number fraction $\phi \left(t\right)$ can be described by solving Equation (6) as

(7) $$\phi \left(t\right)=\frac{\eta_{0}T_{0}I_{0}}{\eta_{0}T_{0}I_{0}+1}+\left(\phi_{0}-\frac{\eta_{0}T_{0}I_{0}}{\eta_{0}T_{0}I_{0}+1}\right)\exp\left[-\frac{t}{T_{0}}\left(\eta_{0}T_{0}I_{0}+1\right)\right],$$

where $\phi_{0}$ refers to the *cis* number fraction at t=0. In the illuminated state, it is considered that $\phi_{0}=0$. Therefore, Equation (7) can be reduced to

(8) $$\phi (t)=\frac{\eta_{0}T_{0}I_{0}}{\eta_{0}T_{0}I_{0}+1}\left\{1-\exp\left[-\frac{t}{T_{0}}\left(1+\eta_{0}T_{0}I_{0}\right)\right]\right\}.$$

In the non-illuminated state, the light intensity $I_{0}=0$, and Equation (7) can be reduced to

(9) $$\phi \left(t\right)=\phi_{0}\exp\left(-\frac{t}{T_{0}}\right),$$

where the initial fraction of *cis* isomers number in the non-illuminated state $\phi_{0}$ is assumed to be the maximum of $\phi \left(t\right)$ in Equation (8), namely $\phi_{0}=\eta_{0}T_{0}I_{0}/\eta_{0}T_{0}I_{0}+1$. Bringing $\phi_{0}=\eta_{0}T_{0}I_{0}/\eta_{0}T_{0}I_{0}+1$ into Equation (9) can yield

(10) $$\phi \left(t\right)=\frac{\eta_{0}T_{0}I_{0}}{\eta_{0}T_{0}I_{0}+1}\exp\left(-\frac{t}{T_{0}}\right).$$

To simplify the calculation, we define the following dimensionless parameters: ${\bar{I}}_{0}=\eta_{0}T_{0}I_{0}$, $\bar{t}=t/T_{0}$, and $\bar{\phi }\left(\bar{t}\right)=\phi \left(\bar{t}\right)\left(\eta_{0}T_{0}I_{0}+1\right)/\eta_{0}T_{0}I_{0}$. Therefore, in the illuminated state, the dimensionless *cis* number fraction $\bar{\phi }\left(\bar{t}\right)$ can be obtained by rewriting Equation (8) in dimensionless form as

(11) $$\bar{\phi }\left(\bar{t}\right)=1-\exp\left[-\bar{t}\left({\bar{I}}_{0}+1\right)\right].$$

In the non-illuminated state, $\bar{\phi }\left(\bar{t}\right)$ can be obtained by rewriting Equation (10) in dimensionless form as

(12) $$\bar{\phi }\left(\bar{t}\right)=\exp\left(-\bar{t}\right).$$

In this paper, the time of one illumination on and illumination off is defined as one illumination period, represented by $T_{L}$. $T_{\mathrm{on}}$ and $T_{\mathrm{off}}$ denote the time of illumination on and illumination off within an illumination period $T_{L}$, respectively. There exists $T_{L}=T_{\mathrm{on}}+T_{\mathrm{off}}$, and $T_{\mathrm{on}}/T_{L}$ is named as the illumination time rate. In addition, the dimensionless forms of $T_{L}$, $T_{\mathrm{on}}$, and ^−^$T_{\mathrm{off}}$ are defined as ${\bar{T}}_{L}=T_{L}/T_{0}$, ${\bar{T}}_{\mathrm{on}}=T_{\mathrm{on}}/T_{0}$, and ${\bar{T}}_{\mathrm{off}}=T_{\mathrm{off}}/T_{0}$, respectively.

**Figure 2:**
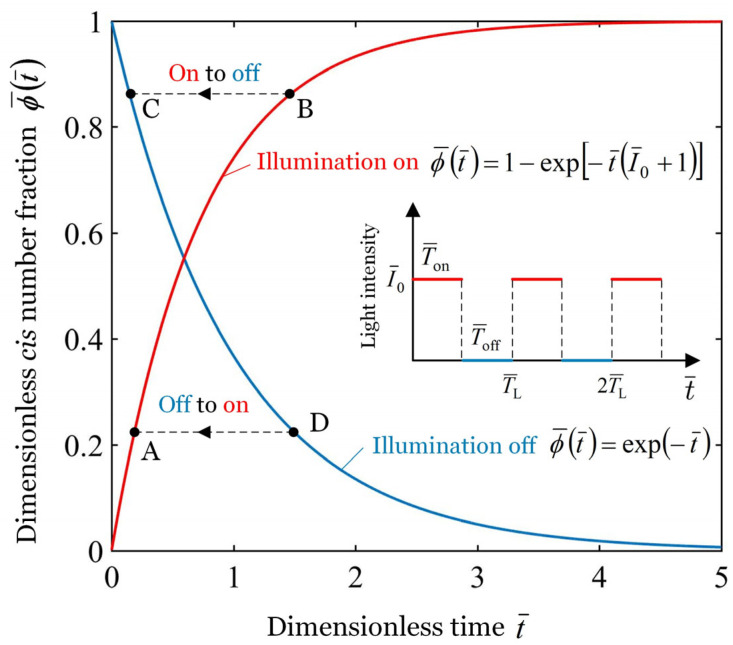
The variation of the dimensionless *cis* number fraction $\bar{\phi }$ as a function of time $\bar{t}$ under a periodic illumination with given light intensity ${\bar{I}}_{0}=0.35$.

As shown in Figure 2, the “Illumination on” and “Illumination off” curves represent the change in the *cis* number fraction with time when the illumination is on and off, respectively. As can be seen from the figure, the dimensionless *cis* number fraction $\bar{\phi }$ increases with the extension of the illumination time and decreases with the passage of the non-illumination time. Specifically, during the first period of illumination ${\bar{T}}_{\mathrm{on}}$, the *cis* number fraction $\bar{\phi }$ increases from 0 to point B along the “Illumination on” curve. Next, transitioning from illuminated state to non-illuminated state, the *cis* number fraction $\bar{\phi }$ shifts from point B to point C. During the first period of non-illumination ${\bar{T}}_{\mathrm{off}}$, the *cis* number fraction $\bar{\phi }$ decreases from point C to point D along the “Illumination off” curve. Then, transitioning from non-illuminated state to illuminated state, the *cis* number fraction $\bar{\phi }$ shifts from point D to point A. During the second ${\bar{T}}_{\mathrm{on}}$, $\bar{\phi }$ rises from point A to point B. In this way, the *cis* number fraction $\bar{\phi }$ switch between the curves of the “Illumination on” and “Illumination off”, and result in light-excited contraction strain $\varepsilon_{L}$ change in periodicity, and finally leads to the steady forced oscillation of the bionic arm. When the periodic illumination is switches between illumination on and illumination off, the evolution of *cis* number fraction $\bar{\phi }$ within the LCE fiber is correspondingly converted between Equations (11) and (12). The study by Nägele et al. [60] demonstrated that the response time of azobenzene isomerization is on the femtosecond scale, which is significantly shorter than the illumination period. Therefore, it can be assumed that the conversion is done in an instant, and the *cis* number fraction $\bar{\phi }$ within the fiber remains unchanged at the instant of conversion.

### 2.3. Governing Equations

To simplify the calculation, the below dimensionless parameters are defined: $\bar{F}\left(t\right)=F\left(t\right)T_{0}^{2}/mL_{2}$, ${\bar{L}}_{1}\left(t\right)=L_{1}\left(t\right)/L_{2}$, ${\bar{L}}_{AB}=L_{AB}/L_{2}$, ${\bar{L}}_{BC}=L_{BC}/L_{2}$, $\bar{c}=cT_{0}/m$, $\bar{g}=gT_{0}^{2}/L_{2}$, and $\bar{k}=kT_{0}^{2}/m$. Bringing Equations (2) and (3) into Equation (1) and then reducing to a dimensionless form yields

(13) $$\bar{\ddot{\theta }}=\bar{g}\cos\theta \left(\bar{t}\right)-\frac{{\bar{L}}_{BC}^{2}+{\bar{L}}_{AB}{\bar{L}}_{BC}\sin\theta \left(\bar{t}\right)}{{\bar{L}}_{1}\left(\bar{t}\right)}\bar{F}\left(\bar{t}\right)-\bar{c}\bar{\dot{\theta }},$$

where $\bar{\ddot{\theta }}=d^{2}\theta /d{\bar{t}}^{2}$ and $\bar{\dot{\theta }}=d\theta /d\bar{t}$. $\bar{F}\left(\bar{t}\right)$ can be derived by taking Equation (3) into Equation (4) and then making it dimensionless as

(14) $$\bar{F}\left(\bar{t}\right)=\bar{k}\left\{\sqrt{{\bar{L}}_{AB}^{2}+{\bar{L}}_{BC}^{2}+2{\bar{L}}_{AB}{\bar{L}}_{BC}\sin\theta \left(\bar{t}\right)}-\left[1+\varepsilon_{L}\left(\bar{t}\right)\right]\sqrt{{\bar{L}}_{AB}^{2}+{\bar{L}}_{BC}^{2}}\right\},$$

in which, the light-excited contraction strain $\varepsilon_{L}$ for the illuminated state and non-illuminated state is $\varepsilon_{L}\left(\bar{t}\right)=C_{0}{\bar{I}}_{0}\left\{1-\exp\left[-\bar{t}\left(1+{\bar{I}}_{0}\right)\right]\right\}/\left({\bar{I}}_{0}+1\right)$ and $\varepsilon_{L}\left(\bar{t}\right)=C_{0}{\bar{I}}_{0}\exp\left(-\bar{t}\right)/\left({\bar{I}}_{0}+1\right)$, respectively.

### 2.4. Solution Method

To obtain the time-dependent rotation angle curve, we initially determine the dimensionless *cis*-isomers number fraction $\bar{\phi }\left(\bar{t}\right)$ of the fiber in the illuminated state and non-illuminated state by analytically solving Equations (11) and (12), as shown in Figure 2. Subsequently, we calculate the light-excited contraction strain $\varepsilon_{L}\left(\bar{t}\right)$ of the fiber by analytically solving Equation (5) based on the obtained *cis*-isomers number fraction $\bar{\phi }\left(\bar{t}\right)$. Next, we numerically solve Equation (14) to determine the spring force $\bar{F}\left(\bar{t}\right)$. Finally, the rotation angle versus time curve is obtained by numerically solving Equation (13). We perform numerically solve Equations (13) and (14) using Matlab 2016b software with classical fourth-order Runge–Kutta method.

Before numerically solving, we first transform Equation (13) into two coupled first-order ordinary differential equations as

(15) $$\left\{\begin{array}{l} \bar{\dot{\theta }}=\bar{\omega }\\ \bar{\dot{\omega }}=f\left(\bar{t},\theta ,\bar{\omega }\right)\\ \bar{\omega }\left({\bar{t}}_{0}\right)={\bar{\dot{\theta }}}_{0}\\ \theta \left({\bar{t}}_{0}\right)=\theta_{0} \end{array}\right..$$

The equation for the fourth-order Runge–Kutta is written as

(16) $$\left\{\begin{array}{l} \theta_{n+1}=\theta_{n}+h{\bar{\omega }}_{n}+\frac{h^{2}}{6}\left(K_{1}+K_{2}+K_{3}\right)\\ {\bar{\omega }}_{n+1}={\bar{\omega }}_{n}+\frac{h}{6}\left(K_{1}+2K_{2}+2K_{3}+K_{4}\right) \end{array}\right.,$$

where

(17) $$\left\{\begin{array}{l} K_{1}=f\left({\bar{t}}_{n},\theta_{n},{\bar{\omega }}_{n}\right)\\ K_{2}=f\left({\bar{t}}_{n}+\frac{h}{2},\theta_{n}+\frac{h}{2}{\bar{\omega }}_{n},{\bar{\omega }}_{n}+\frac{h}{2}K_{1}\right)\\ K_{3}=f\left({\bar{t}}_{n}+\frac{h}{2},\theta_{n}+\frac{h}{2}{\bar{\omega }}_{n}+\frac{h^{2}}{4}K_{1},{\bar{\omega }}_{n}+\frac{h}{2}K_{2}\right)\\ K_{4}=f\left({\bar{t}}_{n}+h,\theta_{n}+h{\bar{\omega }}_{n}+\frac{h^{2}}{2}K_{2},{\bar{\omega }}_{n}+hK_{3}\right) \end{array}\right.,$$

and h represents the time step. In the numerical calculation, we set *h* = 0.001. Using the given initial conditions and the analytical solution of the light-excited contraction strain $\varepsilon_{L}$, the spring force $\bar{F}\left({\bar{t}}_{0}\right)$ at time ${\bar{t}}_{0}$ is found by solving Equation (14). Then, based on $\bar{F}\left({\bar{t}}_{0}\right)$, the angle $\theta \left({\bar{t}}_{1}\right)$ and angular velocity $\bar{\omega }\left({\bar{t}}_{1}\right)$ at time ${\bar{t}}_{1}$ can be obtained by solving Equation (13) using the fourth-order Runge–Kutta method. The angle–time curve of the bionic arm can be plotted by iteration.

## 3. Results and Discussion

From Equations (13) and (14), it can be concluded that the amplitude of the forced oscillation depends on the length from point A to point B ${\bar{L}}_{AB}$, the length from point B to point C ${\bar{L}}_{BC}$, the time of an illumination period ${\bar{T}}_{L}$, the illumination time rate ${\bar{T}}_{\mathrm{on}}/{\bar{T}}_{L}$, the elastic coefficient of the fiber $\bar{k}$, the gravitational acceleration $\bar{g}$, the contraction coefficient of the LCE material $C_{0}$, the light intensity $I_{0}$, and the damping coefficient $\bar{c}$. This section will explain the mechanism of the forced oscillation and quantitatively analyze the influence of these system parameters on the amplitude and equilibrium position of the forced oscillation. Prior to the numerical calculation, we estimated the standard values for the system parameters based on previous studies and empirical data [45,56], as presented in Table 2. According to the values listed in Table 2, it is clear that the overall performance of LCE fibers is better than that of nylon fibers [6] and hydrogel fibers [7,8,9]. For example, LCE fibers have a much faster response speed (0.01~0.1 s) compared to nylon fibers (1~5 s) and hydrogel fibers (10~100 s). The range of values and definitions for each dimensionless parameter derived from the typical values of these parameters are listed in Table 3.

### 3.1. Controllable Forced Vibration of the Bionic Arm

**Figure 3:**
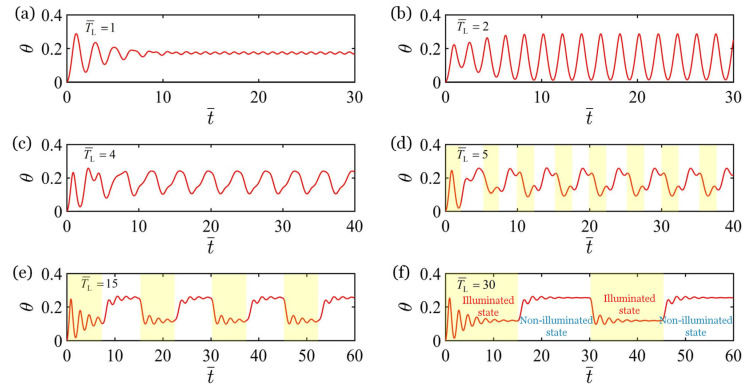
The forced vibration of the bionic arm for six different nondimensionalized illumination periods with given parameters: ${\bar{L}}_{\mathrm{AB}}=1.0$ ${\bar{L}}_{\mathrm{BC}}=0.5$, ${\bar{T}}_{\mathrm{on}}/{\bar{T}}_{L}=0.5$, $\bar{k}=18$, $\bar{g}=1.2$, $C_{0}=-0.28$, ${\bar{I}}_{0}=0.35$, $\bar{c}=0.2$, ${\bar{\dot{\theta }}}_{\bar{t}=0}=0$, and $\theta_{\bar{t}=0}=0$. From (**a**–**f**), ${\bar{T}}_{L}=1$, 2, 4, 5, 15, and 30, respectively. The shaded regions in (**d**–**f**) indicate periods of illumination, the non-shaded regions represent periods of non-illumination. The stable forced vibration period of the system is consistent with the illumination period.

Figure 3 depicts the forced oscillation of the bionic arm for six different nondimensionalized illumination periods, where the parameters are ${\bar{L}}_{\mathrm{AB}}=1.0$, ${\bar{L}}_{\mathrm{BC}}=0.5$, ${\bar{T}}_{\mathrm{on}}/{\bar{T}}_{L}=0.5$, $\bar{k}=18$, $\bar{g}=1.2$, $C_{0}=-0.28$, ${\bar{I}}_{0}=0.35$, $\bar{c}=0.2$, ${\bar{\dot{\theta }}}_{\bar{t}=0}=0$, and $\theta_{\bar{t}=0}=0$. The shaded regions in Figure 3d–f indicate the periods of illumination, while the non-shaded regions represent periods of non-illumination. Consistent with the experimental result [56], the stable forced vibration period of the system is consistent with the illumination period ${\bar{T}}_{L}$. As illustrated in Figure 3, after a short period of unsteady vibration, the system quickly evolved into a stable periodic vibration. Apparently, the initial vibration of the bionic arm consists of both free vibration and light-powered forced vibration. As the vibration persists, the free vibration dissipates rapidly due to damping, leaving only stable forced vibration. Observing Figure 3a-f, it is evident that, with the increase in illumination period ${\bar{T}}_{L}$, the vibration period progressively lengthens, while the amplitude initially increases and subsequently decreases. When the illumination period ${\bar{T}}_{L}\geq 5$, the single-peak steady-state vibration transitions into multi-peak steady-state vibration, and the number of peaks in multi-peak vibration increases first and then remains stable with the increase in the illumination period, as depicted in Figure 3d–f, which is caused by the fact that the illumination period is significantly longer than the natural period of the system. When the illumination period is much longer than the natural period of the system, the system exhibits free oscillation during a period of illumination or a period of non-illumination, and the free oscillation gradually attenuates due to damping, as depicted in Figure 3f. For the ease of analysis, we only consider the case of single-peak steady-state vibration in the subsequent discussion.

### 3.2. Mechanism of the Controllable Forced Vibration of the Bionic Arm

**Figure 4:**
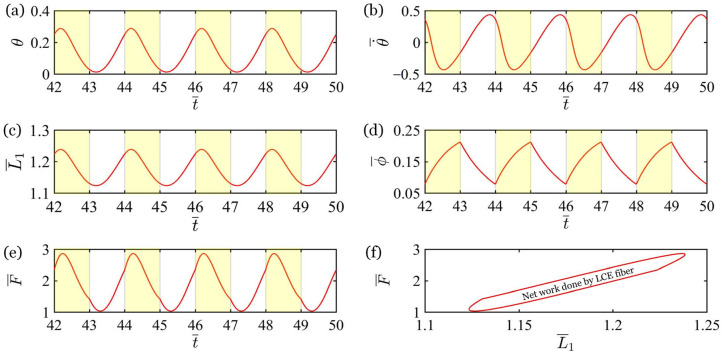
Mechanism of the forced vibration of the bionic arm controlled by periodic illumination. The parameters are ${\bar{L}}_{\mathrm{AB}}=1.0$${\bar{L}}_{\mathrm{BC}}=0.5$, ${\bar{T}}_{L}=2$, ${\bar{T}}_{\mathrm{on}}/{\bar{T}}_{L}=0.5$, $\bar{k}=18$, $\bar{g}=1.2$, $C_{0}=-0.28$, ${\bar{I}}_{0}=0.35$, $\bar{c}=0.2$, ${\bar{\dot{\theta }}}_{\bar{t}=0}=0$, and $\theta_{\bar{t}=0}=0$. (**a**) Time-dependent change in rotation angle θ; (**b**) The relationship between angular velocity $\bar{\dot{\theta }}$ and time; (**c**) Evolution of fiber length ${\bar{L}}_{1}$ over time; (**d**) Number fraction of *cis*-isomers $\bar{\phi }$ change with time; (**e**) Variation in the spring force $\bar{F}$ with time; (**f**) The relationship between the spring force $\bar{F}$ and the length of the fiber ${\bar{L}}_{1}$. The shaded regions in (**a**–**e**) indicate periods of illumination, and the non-shaded regions represent periods of non-illumination.

Figure 4 explores the mechanism of the periodic oscillation of the bionic arm controlled by periodic illumination, and the parameters are ${\bar{L}}_{\mathrm{AB}}=1.0$, ${\bar{L}}_{\mathrm{BC}}=0.5$, ${\bar{T}}_{L}=2$, ${\bar{T}}_{\mathrm{on}}/{\bar{T}}_{L}=0.5$, $\bar{k}=18$, $\bar{g}=1.2$, $C_{0}=-0.28$, ${\bar{I}}_{0}=0.35$, $\bar{c}=0.2$, ${\bar{\dot{\theta }}}_{\bar{t}=0}=0$, and $\theta_{\bar{t}=0}=0$. During the vibration of the bionic arm, the rotation angle θ, the angular velocity $\bar{\dot{\theta }}$, the fiber length ${\bar{L}}_{1}$, the *cis* number fraction $\bar{\phi }$, and the spring force $\bar{F}$ exhibit periodic changes over time, as depicted in Figure 4a–e. The shaded regions and the non-shaded regions represent periods of illumination and periods of non-illumination, respectively. When the bionic arm is illuminated, the *cis* number fraction $\bar{\phi }$ increases. Combining Equation (5), it can be concluded that the light-induced contraction strain $\varepsilon_{L}$ also increases, driving the bionic arm to rotate counterclockwise. Due to the inertia of the mass block, the system’s response is not instantaneous. The rotation angle θ, fiber length ${\bar{L}}_{1}$, and spring force $\bar{F}$ to first increase slightly before decreasing. Meanwhile, the angular velocity $\bar{\dot{\theta }}$ initially decreases, then reverses direction and increases, before finally decreasing again. Conversely, when the bionic arm is in the non-illuminated state, the *cis* number fraction $\bar{\phi }$ and the contraction strain $\varepsilon_{L}$ decreases, driving the bionic arm to rotate clockwise. The rotation angle θ, fiber length ${\bar{L}}_{1}$, and spring force $\bar{F}$ first decrease slightly before increasing. Importantly, when the mass block vibrates upward and downward, the spring force of the fiber passing through the same position is different. Therefore, the spring force of the LCE fiber is in a closed-loop relationship with its length, as depicted in Figure 4e. The net work done by the fiber counteracts the dissipation of the system damping to maintain a stable forced oscillation.

### 3.3. Optimal Illumination Period

**Figure 5:**
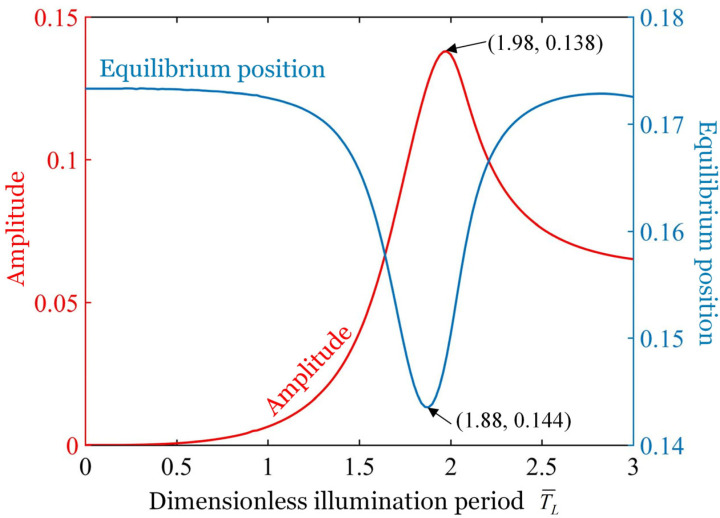
Effect of the dimensionless illumination period on the amplitude and equilibrium position of the oscillation with given parameters ${\bar{L}}_{\mathrm{AB}}=1.0$ ${\bar{L}}_{\mathrm{BC}}=0.5$, ${\bar{T}}_{\mathrm{on}}/{\bar{T}}_{L}=0.5$, $\bar{k}=18$, $\bar{g}=1.2$, $C_{0}=-0.28$, ${\bar{I}}_{0}=0.35$, $\bar{c}=0.2$, ${\bar{\dot{\theta }}}_{\bar{t}=0}=0$, and $\theta_{\bar{t}=0}=0$. An optimal illumination period ${\bar{T}}_{\mathrm{op}}=1.98$ exists that maximizes the amplitude of the oscillation, while a critical illumination period ${\bar{T}}_{\mathrm{cr}}=1.88$ exists that minimizes the equilibrium position of the oscillation.

Figure 5 illustrates the effect of dimensionless illumination period on both the amplitude and the equilibrium position of the vibration, where the parameters ${\bar{L}}_{\mathrm{AB}}=1.0$, ${\bar{L}}_{\mathrm{BC}}=0.5$, ${\bar{T}}_{\mathrm{on}}/{\bar{T}}_{L}=0.5$, $\bar{k}=18$, $\bar{g}=1.2$, $C_{0}=-0.28$, ${\bar{I}}_{0}=0.35$, $\bar{c}=0.2$, ${\bar{\dot{\theta }}}_{\bar{t}=0}=0$, and $\theta_{\bar{t}=0}=0$ are used. An optimal illumination period ${\bar{T}}_{\mathrm{op}}=1.98$ exists that maximizes the amplitude of the vibration, while a critical illumination period ${\bar{T}}_{\mathrm{cr}}=1.88$ exists that minimizes the equilibrium position of the vibration. The figure clearly demonstrates that the amplitude of the vibration initially increases and then decreases as the illumination period ${\bar{T}}_{L}$ increases. Specifically, the forced oscillation achieves a maximum amplitude of 0.138 when the illumination period is 1.98. This is because, when the illumination period is close to the natural period of the system, the resonance is formed and the amplitude is the largest. On the contrary, the equilibrium position of the oscillation initially decreases and then increases as the illumination period ${\bar{T}}_{L}$ increases. The equilibrium position of the forced oscillation reaches a minimum value of 0.144 when the illumination period is 1.88. We can summarize from the energy point of view that when the illumination period is shorter than the system’s natural period, the high-frequency contraction–relaxation cycles of the LCE fiber reduce energy input efficiency, resulting in smaller amplitudes (Figure 3a). In contrast, when the illumination period significantly exceeds the natural period, the system undergoes multiple free oscillations per cycle (Figure 3f), and the damping dissipation increases significantly, leading to a decrease in amplitude. Therefore, only when the illumination period is similar to the natural period of the system, which satisfies the efficient energy input without excess vibration to increase the damping dissipation, should the amplitude be maximized. Based on the findings in this section, the illumination period should be tuned to match the system’s natural period in experiments and applications to obtain larger amplitude and higher efficiency for light energy utilization.

### 3.4. Optimal Illumination Time Rate

**Figure 6:**
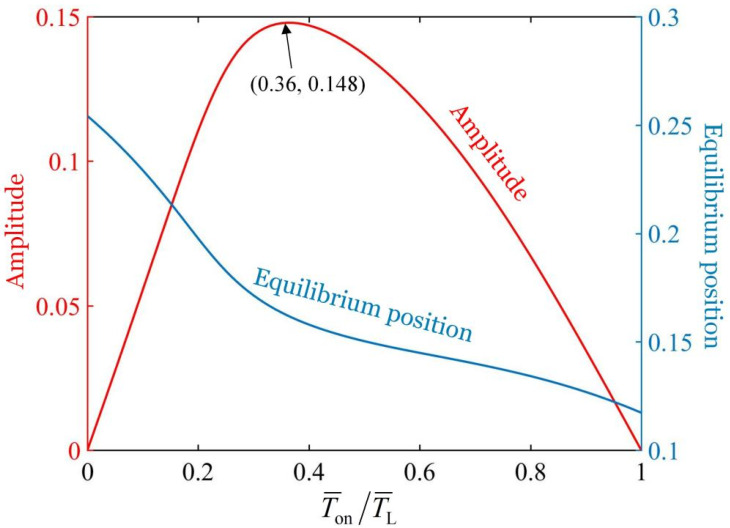
Effect of the illumination time rate on the amplitude and equilibrium position of the oscillation with given parameters ${\bar{L}}_{\mathrm{AB}}=1.0$, ${\bar{L}}_{\mathrm{BC}}=0.5$, ${\bar{T}}_{L}=2$, $\bar{k}=18$, $\bar{g}=1.2$, $C_{0}=-0.28$, ${\bar{I}}_{0}=0.35$, $\bar{c}=0.2$, ${\bar{\dot{\theta }}}_{\bar{t}=0}=0$, and $\theta_{\bar{t}=0}=0$. An optimum illumination time rate ${\bar{T}}_{\mathrm{on}}/{\bar{T}}_{L}=0.36$ can be identified to achieve the maximum amplitude of oscillation.

The variation of the amplitude and equilibrium position of the oscillation with the illumination time rate is depicted in Figure 6, where the parameters ${\bar{L}}_{\mathrm{AB}}=1.0$, ${\bar{L}}_{\mathrm{BC}}=0.5$, ${\bar{T}}_{L}=2$, $\bar{k}=18$, $\bar{g}=1.2$, $C_{0}=-0.28$, ${\bar{I}}_{0}=0.35$, $\bar{c}=0.2$, ${\bar{\dot{\theta }}}_{\bar{t}=0}=0$, and $\theta_{\bar{t}=0}=0$ are used. From the figure, it can be seen that as the illumination time rate ${\bar{T}}_{\mathrm{on}}/{\bar{T}}_{L}$ increases from 0 to 1, the amplitude of the oscillation initially rises from 0 to 0.148 and subsequently declines to 0. There exists an optimal illumination time rate ${\bar{T}}_{\mathrm{on}}/{\bar{T}}_{L}=0.36$ to maximize the amplitude of oscillation. This indicates that both too long and too short illumination times are unfavorable to the oscillation of the bionic arm. Moreover, as the illumination time rate ${\bar{T}}_{\mathrm{on}}/{\bar{T}}_{L}$ increases, the equilibrium position of the oscillation decreases, that is, the equilibrium position moves upward. This phenomenon can be ascribed to the fact that the shorter the period of non-illumination is, the less conducive it is for the fiber to fully recover from the light-excited shrinkage deformation.

### 3.5. Effects of Various Parameters on the Amplitude and Equilibrium Position of the Oscillation

**Figure 7:**
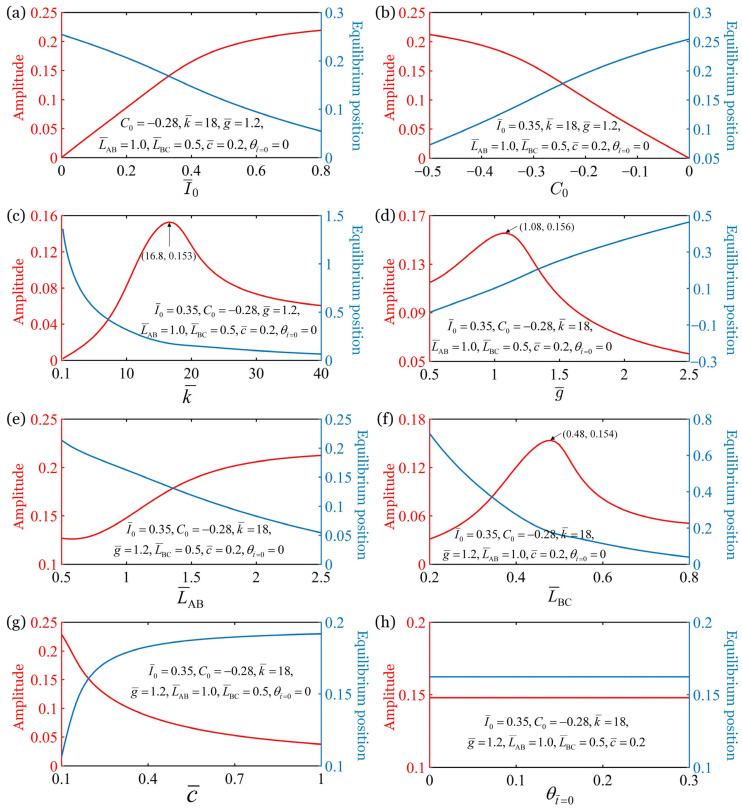
Effects of (**a**) the dimensionless light intensity ${\bar{I}}_{0}$ (**b**) the dimensionless contraction coefficient $C_{0}$, (**c**) the dimensionless elastic coefficient $\bar{k}$, (**d**) the dimensionless gravitational acceleration $\bar{g}$, (**e**) the dimensionless length from point A to point B ${\bar{L}}_{AB}$, (**f**) the dimensionless length from point B to point C ${\bar{L}}_{BC}$, (**g**) the dimensionless damping coefficient $\bar{c}$, and (**h**) the initial rotation angle $\theta_{\bar{t}=0}$ on the amplitude and equilibrium position of the oscillation. The computational parameters are labeled in the figure, and the other parameters are ${\bar{T}}_{L}=2$, ${\bar{T}}_{\mathrm{on}}/{\bar{T}}_{L}=0.36$, and ${\bar{\dot{\theta }}}_{\bar{t}=0}=0$. An optimum elastic constant $\bar{k}=16.6$, gravitational acceleration $\bar{g}=1.08$, and length from point B to point C ${\bar{L}}_{BC}=0.48$ can be identified to achieve the maximum amplitude of oscillation.

This section discusses the effects of the light intensity ${\bar{I}}_{0}$, the contraction coefficient of the LCE material $C_{0}$, the elastic coefficient of the fiber $\bar{k}$, the gravitational acceleration $\bar{g}$, the length from point A to point B ${\bar{L}}_{AB}$, the length from point B to point C ${\bar{L}}_{BC}$, the damping coefficient $\bar{c}$, and the initial rotation angle $\theta_{\bar{t}=0}$ on the amplitude and equilibrium position of the oscillation, as shown in Figure 7. The computational parameters are labeled in the figure, and the other parameters are ${\bar{T}}_{L}=2$, ${\bar{T}}_{\mathrm{on}}/{\bar{T}}_{L}=0.36$, and ${\bar{\dot{\theta }}}_{\bar{t}=0}=0$. There is an optimal elastic coefficient ${\bar{k}}_{\mathrm{op}}=16.6$, gravitational acceleration ${\bar{g}}_{\mathrm{op}}=1.08$, length from point B to point C ${\bar{L}}_{\mathrm{op}}=0.48$ to maximize the amplitude of the forced oscillation of the bionic arm. Figure 7a illustrates that, as the light intensity ${\bar{I}}_{0}$ increases, the oscillation amplitude increases and the equilibrium position decreases monotonically, that is, the equilibrium position moves upwards. This indicates that the greater the light intensity ${\bar{I}}_{0}$, the greater the external excitation to the forced oscillation system, and the more energy the system obtains from the outside. On the other hand, with the increase in the contraction coefficient $C_{0}$ (absolute value decreases), the amplitude decreases and equilibrium position increases, i.e., the equilibrium position of the oscillation moves downwards, as shown in Figure 7b. This suggests that a larger contraction coefficient $C_{0}$ implies a lower utilization rate of light energy. When the contraction coefficient is greater than 0 ($C_{0}>0$), the fiber exhibits light-excited expansion, and the system remains static. Therefore, in practical experimental implementations and applications, the oscillation amplitude and light-energy utilization efficiency of the model can be enhanced by increasing the contraction coefficient of the LCE material through methods, such as elevating the azobenzene content.

The analysis of Figure 7c reveals a notable trend that with the increase in the elastic constant of the fiber $\bar{k}$, there is an initial rise and then decline in the amplitude of the oscillation. Thus, there exists an optimal elastic constant ${\bar{k}}_{\mathrm{op}}=16.8$ that maximizes the amplitude of the bionic arm, and the maximum amplitude is 0.153. Additionally, the equilibrium position consistently decreases as the elastic constant $\bar{k}$ increases. This can be attributed to the natural period of the system decreases with the increase in the elastic constant $\bar{k}$, when the natural period of the system is close to the illumination period, the resonance is formed and the amplitude is the largest. Furthermore, when the dimensionless gravitational acceleration $\bar{g}$ increases, the amplitude of the oscillation increases first and then decreases, and the equilibrium position increases monotonously, as shown in Figure 7d. There exists an optimal gravitational acceleration ${\bar{g}}_{\mathrm{op}}=1.08$ that maximizes the amplitude of the bionic arm, and the maximum amplitude is 0.156. This phenomenon is also a result of the change in the natural period of the system.

Figure 7e illustrates the effect of length from point A to point B ${\bar{L}}_{AB}$ on the amplitude and equilibrium position. The results indicate that, as the ${\bar{L}}_{AB}$ increases, the oscillation amplitude monotonically increases while the equilibrium position monotonically decreases. It can be understood in combination with Figure 1b that $\cos\alpha \left(t\right)$ increases with the increase in length from point A to point B, leading to an increase in the component of the spring force perpendicular to the forearm. From Figure 7f, it can be seen that, as the length from point B to point C ${\bar{L}}_{BC}$ increases, the oscillation amplitude initially increases and then decreases, while the equilibrium position decreases monotonically. There exists an optimal length from point B to point C ${\bar{L}}_{\mathrm{op}}=0.48$ that maximizes the amplitude of the bionic arm, and the maximum amplitude is 0.154. This is caused by the decrease in $\cos\alpha \left(t\right)$ when the length from B to C increases. Therefore, in practical experimental implementations and applications, the fiber should be secured near the midpoint of the forearm to maximize the amplitude of the model.

When the damping coefficient $\bar{c}$ increases, the oscillation amplitude decreases and the equilibrium position increases, as shown in Figure 7g. This implies that the larger the damping coefficient $\bar{c}$ is, the more the oscillation is suppressed. The computational results in Figure 7h show that the amplitude and equilibrium position of the oscillation remain constant with the increase in the initial angle $\theta_{\bar{t}=0}$, indicating that the initial conditions do not affect the amplitude and equilibrium position of the stable oscillation.

## 4. Conclusions

LCE is an advanced multifunctional material that combines the flexibility of polymer networks with the nematic structure of liquid crystals. It has the advantages of a fast response, large deformation, and high energy efficiency, which makes it more suitable for use as an artificial muscle in bionic structures than nylon, hydrogels, and piezoelectric materials. To broaden the application of LCE material in the field of bionic machines and provide theoretical support for the design of LCE-based bionic machines, we established a dynamic model of a bionic arm. The arm was composed of a brachium, a forearm, a joint, a mass block, and an LCE fiber as artificial muscle, which can be periodically oscillation-controlled by periodic illumination. Based on the assumption of linear elasticity model, the periodic spring force of the fiber was given. Subsequently, the evolution equations for the *cis* number fraction within the fiber were developed, and consequently, the analytical solution for the light-excited strain was derived. Following that, the dynamics equation was numerically solved, and the mechanism of the controllable oscillation was elucidated. Numerical calculations show that the stable oscillation period of the bionic arm depends on the illumination period. The results show that the net work done by the fiber counteracts the dissipation of the system damping, maintains a stable forced oscillation, and the stable forced oscillation period of the bionic arm depends on the illumination period.

Furthermore, the effects of various parameters on the amplitude and equilibrium position of the system were investigated. The numerical calculations show that the increases in the light intensity ${\bar{I}}_{0}$, the length from point A to point B ${\bar{L}}_{AB}$ can induce an increase in the amplitude of the bionic arm. Conversely, increasing the system parameters including the contraction coefficient $C_{0}$, the damping coefficient $\bar{c}$ can decrease the amplitude. Moreover, the effect of the illumination period ${\bar{T}}_{L}$, the elastic coefficient $\bar{k}$, the gravitational acceleration $\bar{g}$, the illumination time rate ${\bar{T}}_{\mathrm{on}}/{\bar{T}}_{L}$, and the length from point B to point C ${\bar{L}}_{BC}$ on the amplitude is initially increasing and then decreasing. When the illumination period and the natural period of the system are close to each other, the resonance is formed and the amplitude is the largest. The equilibrium position of the system increases monotonically with the increase in $\bar{g}$, $\bar{c}$, $C_{0}$, decreases monotonically with the increase in ${\bar{I}}_{0}$, $\bar{k}$, ${\bar{L}}_{AB}$, ${\bar{L}}_{BC}$, ${\bar{T}}_{\mathrm{on}}/{\bar{T}}_{L}$, and initially increases and then decreases with the increase in ${\bar{T}}_{L}$. The bionic arm using an LCE fiber as artificial muscle has the advantages of simple structure, no need for electronics, ambient power, and rapid response, all of which make it highly practical in the design of micro-machines, bionic devices, soft robots, biomedical devices, and energy harvesters. However, limited by the load-bearing capacity of the LCE fiber, the bionic arm is only suitable for light-load scenarios, and the load-bearing capacity of the bionic arm can be significantly expanded by improving the material properties and optimizing the structure in future research.

## Figures and Tables

**Table 1:** Dimensions and mass of each component of the bionic arm.

Parameter	Definition	Value	Unit
$L_{0}$	Original length of the LCE fiber	10~30	mm
$d_{\mathrm{fi}}$	Diameter of the fiber	500	um
$\rho_{\mathrm{fi}}$	Density of the fiber	1.3	g/cm^3^
$m_{\mathrm{fi}}$	Mass of the fiber	2.6~7.8	mg
$L_{2}$	Straight-line distance between the center of mass and point B (Length of the forearm)	5~20	mm
$A_{\mathrm{fo}}$	Cross-sectional area of the forearm	4	mm^2^
$\rho_{\mathrm{fo}}$	Density of the forearm (in balsa wood)	0.25	g/cm^3^
$M_{\mathrm{fo}}$	Mass of the forearm	0.005~0.02	g
m	Mass of the mass block	2~5	g
$L_{AB}$	Length from point A to point B	10~25	mm
$L_{BC}$	Length from point B to point C	0~20	mm

**Table 2:** Standard values for the system parameters.

Parameter	Definition	Value	Unit
$I_{0}$	Light intensity	0~50	kW/m^2^
$C_{0}$	Contraction coefficient of LCE material	−0.5~0	/
$\eta_{0}$	Light-absorption constant	0.0002	m^2^/s·W
$T_{0}$	Time of thermal relaxation	0.01~0.1	s
$T_{L}$	Time of an illumination period	0~3	s
$T_{\mathrm{on}}$	Time of illumination on	0~3	s
$T_{\mathrm{on}}/T_{L}$	Illumination time rate	0~1	/
$T_{\mathrm{off}}$	Time of illumination off	0~3	s
g	Gravitational acceleration	9.8	m/s^−2^
k	Elastic coefficient of the fiber	1~4	N/m

**Table 3:** Dimensionless parameters.

Dimensionless Parameter	Definition	Value
${\bar{L}}_{AB}$	$L_{AB}/L_{2}$	0.5~5
${\bar{L}}_{BC}$	$L_{BC}/L_{2}$	0~1
${\bar{T}}_{L}$	$T_{L}/T_{0}$	0~30
${\bar{T}}_{\mathrm{on}}$	$T_{\mathrm{on}}/T_{0}$	0~30
$\bar{t}$	$t/T_{0}$	/
$\bar{k}$	$kT_{0}^{2}/m$	0.1~40
$\bar{g}$	$gT_{0}^{2}/L_{2}$	0.2~5
$C_{0}$	/	−0.5~0
${\bar{I}}_{0}$	$\eta_{0}T_{0}I_{0}$	0~1
$\bar{\phi }$	$\phi \left(\eta_{0}T_{0}I_{0}+1\right)/\eta_{0}T_{0}I_{0}$	0~1

## Data Availability

The raw data supporting the conclusions of this article will be made available by the authors upon request.
